# Longitudinal Stability in Reading Comprehension Is Largely Heritable from Grades 1 to 6

**DOI:** 10.1371/journal.pone.0113807

**Published:** 2015-01-20

**Authors:** Brooke Soden, Micaela E. Christopher, Jacqueline Hulslander, Richard K. Olson, Laurie Cutting, Janice M. Keenan, Lee A. Thompson, Sally J. Wadsworth, Erik G. Willcutt, Stephen A. Petrill

**Affiliations:** 1 Department of Psychology, The Ohio State University, Columbus, Ohio, United States of America; 2 Department of Psychology and Neuroscience, University of Colorado Boulder, Boulder, Colorado, United States of America; 3 Institute for Behavioral Genetics, University of Colorado Boulder, Boulder, Colorado, United States of America; 4 Department of Special Education, Psychology, Radiology, and Pediatrics, Vanderbilt University, Nashville, Tennessee, United States of America; 5 Department of Psychology, University of Denver, Denver, Colorado, United States of America; 6 Department of Psychological Sciences, Case Western Reserve University, Cleveland, Ohio, United States of America; The National Institutes of Health, UNITED STATES

## Abstract

Reading comprehension is a foundational academic skill and significant attention has focused on reading development. This report is the first to examine the stability and change in genetic and environmental influences on reading comprehension across Grades 1 to 6. This developmental range is particularly important because it encompasses the timespan in which most children move from learning how to read to using reading for learning. Longitudinal simplex models were fitted separately for two independent twin samples (*N* = 706; *N* = 976). Results suggested that the shared environment contributed to variance in early but not later reading. Instead, stability in reading development was largely mediated by continuous genetic influences. Thus, although reading is clearly a learned skill and the environment remains important for reading development, individual differences in reading comprehension appear to be also influenced by a core of genetic stability that persists through the developmental course of reading.

## Introduction

Reading comprehension, defined as the ability to understand and employ text for learning, is a foundational skill for academic and occupational success. Reading comprehension is clearly a learned skill, subject to differences in instruction and in the environment [1, 2]. Additionally, neurobiological studies suggest that reading comprehension is influenced by individual differences in brain structure and function [3] and ongoing molecular genetic studies continue to examine numerous regions of the genome [4, 5].

The larger reading literature has been engaged in intense study of the relation between early reading, when children acquire reading skills, and later reading, when children are using reading to learn and comprehend. Because the cognitive demand shifts and pedagogic emphases change dramatically in reading over the course of the elementary school years, this transition has been widely referred to in the literature as two stages: “learning to read” and “reading to learn”[6]. In the “learning to read” years there is much direct instruction which focuses on language and decoding skills, whereas the “reading to learn” years primarily consist of independent reading and focus on comprehension. Though reading appears to be very different across the early grades, as the task of reading increasingly shifts away from decoding words towards understanding and using text for learning, there is also evidence pointing towards stability in reading ability. For example, several aspects of early reading ability involved in “learning to read” such as phonological awareness [7], decoding [8], fluency [9], and vocabulary [10] predict successful reading comprehension across time, including while “reading to learn.” Juel [11] found that reading ability at first grade was highly predictive of reading in fourth grade. Similarly, reading comprehension from first through fifth grades showed a pattern of stability in individual differences across the five years of reading development [12]. Stability in reading comprehension is also observed for those with reading difficulties [13, 14]. Thus, though the demands of reading appear very different across early years of reading, there is also evidence of stability in reading ability.

Given the findings from phenotypic research, a central question is how genetic and environmental factors influence the etiology and longtitudinal stability and instability of individual differences in reading comprehenion. Behavioral genetic studies build upon phenotypic studies by offering insight into the genetic and environmental etiologies underlying the phenotypic relations. The study of the etiology of reading has a long history, beginning with the study of the etiology of the diagnosis of reading disability [15], shifting to the study of reading-related cognition [16, 17], and more recently has included studies of individual differences in reading comprehension [18, 19]. The vast majority of these studies have examined word-level decoding, or reading-related skills such as phonological awareness, print awareness, fluency, and vocabulary [20–22]. In general, these studies suggest that genetics and the shared environment are important in preschool and in early school. However, after the initiation of formal schooling, shared environmental effects decrease and often become nonsignificant, leaving high heritabilities and the nonshared environment as the primary sources of variability in reading [23–25].

Beyond the univariate question of “whether” reading comprehension is influenced by genetic and environmental factors, a more important issue is the genetic and environmental contribution to the longitudinal stability and instability of reading comprehension, particularly as the demands of reading shift. Reading-related skills key to early reading success and predictive of later reading ability show both distinct genetic influences and overlap of genetic effects on reading [26]. There have been some longitudinal behavioral genetic studies investigating overlap of etiological influences on individual differences in reading, but they have been limited to relatively narrow developmental windows, for example, preschool to grade 2 [26], ages 6 to 7 [21], and ages 7 to 10 [23]. These studies tend to show both overlap and some independent genetic effects. More recently, Hart and colleagues [24] examined oral reading fluency development from grades 1 to 5. Genetic influences were largely stable, though there were smaller but still significant novel genetic effects coming online in grades 1 to 3 which encompass the “learning to read” years. Moderate shared environmental influences were entirely continuous, while nonshared environmental influences contributed to both the continuity and change in individual differences in oral reading fluency. This general pattern is consistent with other cognitive outcomes, in particular general cognitive ability, see [27, 28].

However, no genetically sensitive study to date has examined the genetic and environmental contributions to the stability of reading comprehension, outside of a study that showed genetic stability in very small (1-year) developmental window [21]. Reading comprehension is a particularly important outcome given that it is the ultimate goal of reading and much academic achievement is dependent upon it. In addition to focusing on reading comprehension rather than single word reading, the current study expands upon previous longitudinal work in two important and novel ways. First, the use of genetically-sensitive simplex modeling [29] allows us to explore the extent to which individual differences in reading comprehension over time are influenced by stable genetic and environmental factors and/or by genetic and environmental factors that emerge at different time points along development. The simplex model is the most appropriate model to address continuity of influences on reading in that it directly estimates the stability of reading over time by regressing later reading achievement onto earlier measures of reading achievement. The autoregressive nature of the model allows for quantification of across-time within-construct (here, reading) stability while also quantifying additional time-specific influences. Second, we have reading comprehension data from both the Western Reserve Reading and Math Project (WRRMP) and the International Longitudinal Twin Study (ILTS). By fitting the models to two independent twin samples, we are able to observe the extent to which findings replicated from one sample to the other.

Thus, the purpose of the current study was to examine the genetic and environmental contributions to the stability and instability of reading comprehension from Grade 1 to Grade 6 (WRRMP) and Grade 1 to Grade 4 (ILTS), using both Western Reserve Reading and Math Project (WRRMP) and International Longitudinal Twin Study (ILTS) samples. The larger reading literature suggests we should expect some longitudinal stability in reading comprehension despite the fact that the relative task demands of reading change as reading is acquired. While previous behavioral genetic results show that reading comprehension is highly heritable, particularly after the first year of formal reading instruction, there are several possibilities concerning the role of genetic and environmental influences in the stability and instability of reading comprehension. First, genes may influence a core of reading ability that remains largely stable despite changes in task demands as reading is acquired. On the other hand, genetic influences may also change, reflecting change in task demands or changes in the cognitive skills necessary for reading. Moreover, child-specific experiences from the nonshared environment may also contribute to longitudinal stability or change as reading comprehension changes. Finally, shared environment influences may also be important to stability in early reading, even though they may not be significant in later reading.

## Method

### Participants

**Western Reserve Reading and Math Project (WRRMP) Sample.** The Western Reserve Reading and Math Project [30] is an ongoing longitudinal unselected twin study in Ohio investigating reading, math, and related cognitive skills. Twins were recruited through schools, media advertisements, Ohio state birth records, and twin clubs. Additionally, a community social worker was hired specifically to aid in the recruitment of underrepresented groups [31].

Participants were 292 monozygotic (MZ; 58.22% female) and 414 same-sex dizygotic (DZ; 55.07% female) twin children who participated in testing visits 1–8 of the WRRMP. Though the large majority of twins entered the study in testing visit 1, additional participants were recruited subsequently. Data collection for WRRMP is ongoing and families are tested throughout the calendar year. Current growth analyses were conducted based on the grade the children were in at each testing visit. The twins were approximately 7 years old (*M* age at Grade 1 = 7.10, *SD* = 0.45) in first grade. The number of twins with Reading Comprehension scores for each grade is provided in Table 1.

**Table 1:** Descriptive Statistics, Intraclass Correlations, and Univariate Genetic (a²), Shared Environmental (c²), and Nonshared Environmental (e²) Components of Variance for Reading Comprehension.

	**Descriptive Statistics**					**Intraclass Correlations**				**Univariate**		
						**MZ**		**DZ**				
**Variable**	**Mean**	**SD**	**Min.**	**Max.**	***n***	***r***	***n***	***r***	***n***	**a^2^**	**c^2^**	**e^2^**
Reading Comp. WRRMP												
Grade 1	107.98	10.09	62	136	448	0.80	178	0.52	270	0.57* [.37, .76]	0.24* [.07, .41]	0.19* [.14, .25]
Grade 2	110.46	9.93	74	147	406	0.72	168	0.46	238	0.52* [.31, .71]	0.18* [.00, .40]	0.30* [.24, .38]
Grade 3	107.96	10.43	58	131	399	0.80	171	0.35	228	0.75* [.60, .82]	0.02 [.00, .16]	0.22* [.17,.29]
Grade 4	105.72	10.61	73	129	367	0.75	152	0.33	215	0.73* [.55, .80]	0.02 [.00, .20]	0.26* [.20, .33]
Grade 5	105.94	10.78	78	137	378	0.75	153	0.27	225	0.77* [.52,.83]	0.00 [.00, .23]	0.22* [.17, .30]
Grade 6	103.63	10.67	65	125	243	0.74	93	0.35	150	0.74* [.46, .81]	0.01 [.00, .28]	0.26* [.19, .34]
Reading Comp. ILTS												
Grade 1	104.80	12.84	58	140	960	0.78	440	0.47	518	0.68* [.52,.82]	0.12 [.00, .26]	0.20* [.17, .24]
Grade 2	100.12	12.13	41	138	964	0.69	442	0.46	522	0.58* [.41, .73]	0.14* [.01, .29]	0.28* [.24, .33]
Grade 4	98.52	13.78	43	142	928	0.71	418	0.39	510	0.70* [.50, .76]	0.02 [.00, .20]	0.28* [.23, .33]

*Note. N*s are for individuals.

*p <.05; 95% confidence intervals are in brackets.

Twin zygosity was determined by DNA analyses on samples collected via buccal swab. In a minority of cases (*N* = 76), parents opted to complete a questionnaire based on twins’ physical similarity to determine zygosity [32], a method which has been used in other studies with 95% accuracy when compared to DNA analyses [33].

**International Longitudinal Twin Study (ILTS) Sample.** The International Longitudinal Twin Study [26] is an ongoing longitudinal unselected twin study investigating reading and related skills in Colorado, Australia, and Scandinavia. The current study includes only the Colorado sample, for which twins were recruited through state birth records.

Participants were 446 monozygotic (MZ; 56.50% female) and 530 same-sex dizygotic (DZ; 44.91% female) twin children. The twins were approximately 7 years old (*M* age = 7.42, *SD* = 0.32) at the end of first grade. The number of twins with Reading Comprehension scores for each grade is provided in Table 1.

Zygosity was determined from DNA extracted from cheek swabs, or in a minority of cases (16%) from selected items from the Nichols and Bilbro [34] questionnaire.

### Ethical statement

Ethical approval for the WRRMP study has been provided by The Ohio State University Institutional Review Board (reference: 2006B0291). The parents of the twins provided informed written consent/permission for each assessment. Ethical approval for the ILTS study has been provided by Institutional Review Board University of Colorado-Boulder (reference: FWA00003492). The parents of the twins provided informed written consent/permission for each assessment.

### Procedure

**WRRMP Sample.** Data were collected approximately annually for a total of six home visits. Twins were assessed on all measures in separate rooms in their homes by trained testers. Each test session lasted approximately three hours per child.

**ILTS Sample.** Data for the current study were collected in the summers following the first-, second-, and fourth-grades. Twins were assessed separately in their homes by trained testers. Each test session lasted approximately one hour.

### Measure

Reading Comprehension, the focus of the current study, was assessed using the Woodcock Reading Mastery Test-Revised (WRMT-R) [35] Passage Comprehension subtest for both the WRRMP and ILTS samples. This measure consists of short passages (one to three sentences long) which are missing a key word. As the task progresses, passages increase in difficulty with regard to characteristics such as length, decoding, and semantics. The participant is required to read the passages and produce missing words. The task continues until the participant is no longer able to supply the missing words correctly. This measure has a published split-half reliability of.73 to.94. Within each sample, all reading scores were corrected for age and sex using the regression technique as outlined by McGue and Bouchard [36] prior to conducting twin analyses to control for potential age and sex effects.

### Analyses

All analyses were conducted separately for the WRRMP and ILTS samples. Parallel analyses allowed for comparison of the pattern of results across the two independent longitudinal samples. This within-study replication design provided a test of the generalizability of findings.

**Phenotypic analyses.** As a first step, descriptive statistics for each year of Reading Comprehension were calculated for both the WRRMP and ILTS samples. Then, phenotypic correlations for each sample were examined among all years to quantify the strength of the relation from year to year.

**Behavioral genetic analyses.** The genetic and environmental influences on reading ability were first assessed through examination of twin intraclass correlations. Because MZ twins share the same genotype and the same shared environment, whereas DZ twin share only half of their segregating genes (i.e., the genes that serve to make individuals different from one another), on average, comparison of MZ and DZ intraclass correlations offers insights into the expected influences of genetic and environmental influences. Due to the differences in shared genotype in MZ versus DZ twins, if MZ twins are more similar than DZ twins, genetic influences are implied. Shared environmental effects are suggested to the extent that MZ and DZ correlations are similar in magnitude. Nonshared environmental effects (and measurement error) are implied by the difference between MZ twins’ correlation and a perfect correlation (1.00).

A quantitative genetic simplex model [29] was estimated to examine the stable and age-specific genetic and environmental factors underpinning the relations across the six or three annual assessments of reading development for WRRMP and ILTS samples, respectively. Models were fit to all available data using the Mx structural equation modeling program [37] which handles missing outcome variables (i.e., Reading Comprehension) using full-information maximum likelihood. Specifically, the variance across each of the grades of Reading Comprehension was simultaneously decomposed into continuous and novel effects of genetic variance (A; inherited additive genetic influences), shared environmental variance (C; environmental influences that cause siblings to be more similar), and nonshared environmental variance (E; environmental influences unique to the individual, as well as error). The importance of continuous (transmitted) and novel (age-specific innovation) effects are assessed by the statistical significance of the respective path estimates (i.e., the model’s quantification of these continuous and novel influences at each grade of reading). Path estimates quantifying continuous and novel influences on reading were identified as statistically significant if their 95% confidence intervals (CI) did not include zero.

Univariate estimates of the proportion of variance due to genetic (*a* ^2^), shared environmental (*c* ^2^), and nonshared environmental influences (*e* ^2^) were estimated for each grade of Reading Comprehension using the simplex model path estimates (see Table 1). Specifically, these univariate estimates are derived as follows: first grade Reading Comprehension, the first time point in the model, is entirely due to age-specific innovation. Thus, the proportion of each etiological type of variance (*a* ^2^, *c* ^2^, and *e* ^2^) for first grade is the squared age-specific innovation path estimate to the respective latent influence (A, C, and E) for that time point. Calculation of proportions of genetic and environmental variance at each subsequent grade is the sum of 1) the proportion of variance (*a* ^2^, *c* ^2^, or *e* ^2^) from the prior grade multiplied by the square of the respective transmission path estimate, and 2) the squared age-specific innovation path estimate (an example is provided below).

## Results

### Phenotypic analyses

Descriptive statistics are provided in Table 1. Though the raw scores (corrected for age and sex) were used to fit the simplex models, the means and standard deviations for the standard scores are presented in Table 1 as they can be more meaningfully compared to the standardizing population compared to raw scores. The intraclass correlations presented in Table 1 showed greater similarity for MZ than DZ twins, which suggested genetic influences on Reading Comprehension at each grade. Phenotypic correlations presented in Table 2 showed significant relations among all time points. These correlations showed a simplex trend such that the strength of relation was weaker the further apart time points were. Additionally, the strength of the relation between Reading Comprehension from grade to grade tended to increase over time. For example, WRRMP Grade 1 to Grade 2 *r* = .60, whereas there was an increase later from Grade 5 to Grade 6 *r* = .76.

**Table 2:** Phenotypic Correlations for all Grades of Reading Comprehension.

	**Grade 1**		**Grade 2**		**Grade 3**		**Grade 4**		**Grade 5**		**Grade 6**
Reading Comp. WRRMP											
Grade 1	—										
Grade 2	0.60	(262)	—								
Grade 3	0.53	(240)	0.74	(226)	—						
Grade 4	0.58	(236)	0.64	(202)	0.79	(231)	—				
Grade 5	0.49	(235)	0.51	(208)	0.77	(218)	0.82	(243)	—		
Grade 6	0.45	(136)	0.62	(155)	0.71	(111)	0.69	(117)	0.76	(167)	—
Reading Comp. ILTS											
Grade 1	—										
Grade 2	0.78	(952)	—								
Grade 4	0.65	(912)	0.73	(918)							

*Note. N*s in parentheses are for individuals. All correlations are significant at *p* <.001

### Behavioral genetic analyses

Univariate estimates were calculated from the simplex model as described above for both WRRMP and ILTS samples from their respective simplex models. For example, the heritability for WRRMP Grade 1 was calculated as the squared age-specific innovation path to A1 (Grade 1 *a* ^2^ = .756^2^, or .57). The heritability for WRRMP Grade 2 is the sum of the transmission of genetic effects from Grade 1 ([Grade 1 *a* ^2^] × [Grade 1 → Grade 2 transmission]^2^, or .57 × .84^2^) and the age-specific innovations (.35^2^; total Grade 2 *a* ^2^ = [.57 × .84^2^] + [.35^2^] = .52). Univariate estimates for each grade of reading comprehension for both WRRMP and ILTS samples showed substantial genetic influences, modest shared environmental influences, and moderate nonshared environmental influences (see Table 1).

**Figure 1:**
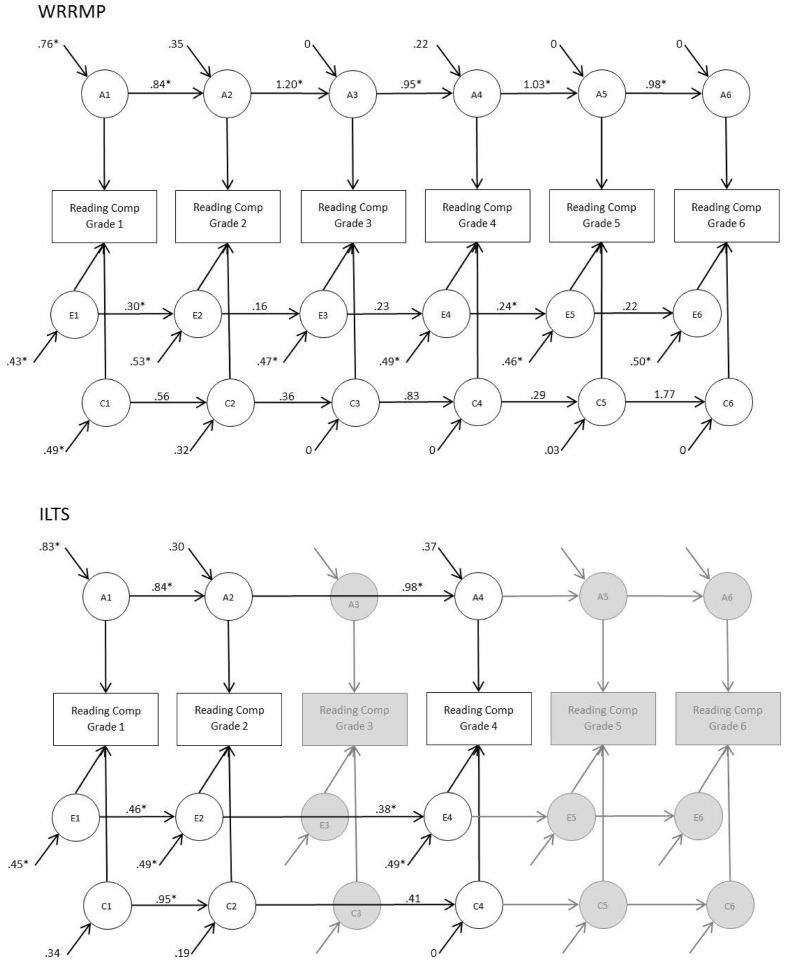
Quantitative genetic simplex model of Reading Comprehension from Grades 1–6. Continuous transmitted and age-specific novel effects are decomposed into genetic (A), shared environmental (C), and nonshared environmental (E) sources. Continuous transmitted effects are quantified by paths from one time point to the next (e.g., the path from A1 → A2 (.84) quantifies the genetic effects transmitted from Reading Comprehension in Grade 1 to Reading Comprehension in Grade 2). Age-specific novel effects are quantified by the disturbances on each latent etiological variable (e.g., the .35 disturbance path onto A2 is the age-specific genetic effect for Reading Comprehension in Grade 2). *p <.05.

**Figure 2:**
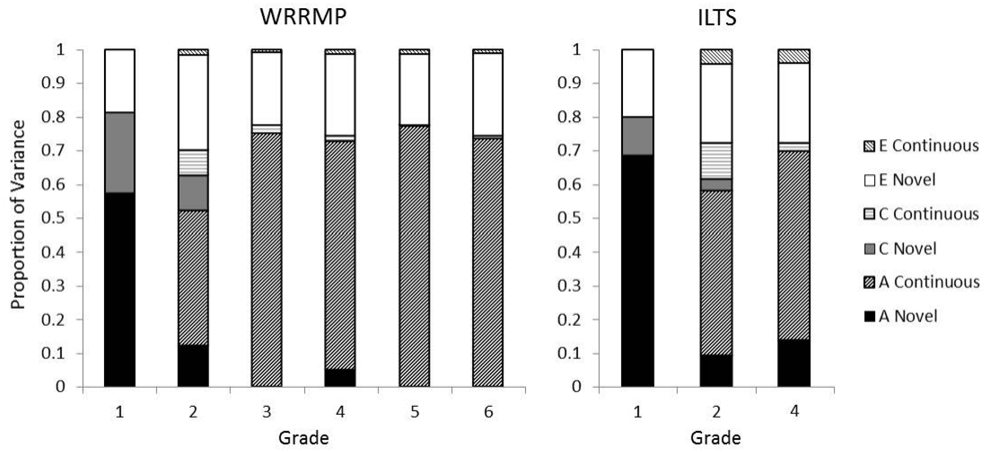
Proportions of variance in Reading Comprehension due to age-specific novel and continuous transmitted sources. Variance is decomposed into genetic (A), shared environmental (C), and nonshared environmental (E) effects. Age-specific novel effects are represented by the solid portions of the bars and continuous transmitted effects are represented by the line-shaded portions of the bars.

The simplex models for the WRRMP and ILTS samples showed remarkably similar patterns of results. For both samples, genetic stability was statistically significant across all grades, as can be seen by the significant transmission paths (i.e., transmission path estimates with 95% CIs not including zero) from each grade to the next (see Fig. 1). The stability of the genetic effects is also clearly seen in Fig. 2 which shows that the transmitted genetic effects (i.e., the line-shaded portion of the bars) accounted for nearly all of the genetic influences across grades. Age-specific genetic innovations were statistically significant only in Grade 1; all subsequent genetic innovations were estimated at zero or were modest and not statistically significantly different from zero. Shared environmental influences on Reading Comprehension came online in Grade 1 and, to a lesser extent, Grade 2 and then were continuous and mostly nonsignificant (see Fig. 1). The modest shared environmental influences at each grade of Reading Comprehension and the proportion which is due to transmitted versus innovative effects can be seen most clearly in Fig. 2. In contrast to genetic and shared environmental influences, nonshared environmental influences showed some significant innovations and transmissions (see Fig. 1), though overall nonshared environmental effects (including measurement error) were primarily novel (see Fig. 2).

## Discussion

The primary aim of this study was to examine the longitudinal stability of genetic and environmental influences on reading comprehension from Grades 1 to 6 and, further, to test the generalizability of findings by investigating two longitudinal twin samples. Genetic influences on reading comprehension present at Grade 1 were largely continuous across all six years of reading development in the WRRMP and all four years (three testing visits) in the ILTS. Very small novel genetic influences tended to come online in early grades when children were “learning to read.” These early elementary school grades typically include direct instruction in reading and novel sources of genetic variance may reflect children’s continued acquisition of reading skill. However, these novel genetic influences were not significant. There tended to be no novel genetic influences in later grades when the focus of literacy education shifts away from direct instruction and more towards the content of what is read, that is, when children are “reading to learn.” Thus, though reading appears to be very different across grades, it is largely influenced by the same genetic factors. Shared environmental influences were small and followed the same pattern of stability across years. Finally, nonshared environmental influences (including measurement error) contributed to instability, or change, across reading development.

Overall, current findings of genetic and environmental stability were similar to those of other constructs, including general cognitive ability [27, 28] and oral reading fluency [24]. Considering the high degree of genetic overlap among different learning outcomes [38], it is plausible that current results for reading comprehension reflect genetic and environmental influences that are more domain-general effects that reflect learning ability generally rather than domain-specific to reading.

On the other hand, there is evidence that general cognitive ability does not account for all of the variance in reading. For example, additional variance is explained by decoding and listening comprehension [19], the two components successful reading comprehension according to the Simple View of Reading [39]. Additionally, other behavioral genetic studies on reading and related skills have suggested some skill-specific effects in addition to continuity of etiological influences. For example, Byrne and colleagues [26] examined several reading and reading-related measures (print awareness, phonological awareness, rapid naming, word reading, reading comprehension, and spelling) and found large common genetic influences as well as additional independent genetic factors. In addition, within the reading domain, both common and independent genetic factors for word recognition and reading comprehension have been observed in a cross-sectional sample of twins with a mean age of 11 years [19]. Another study by Harlaar and colleagues examined teacher-rated reading achievement at ages 7, 9, and 10 and showed substantial genetic overlap across years, but also significant independent genetic factors coming online at both 9- and 10-year old reading [23]. These ages would approximately correspond to grades 1, 3, and 4 in the current study, when very small novel genetic influences come online but do not reach significance. Thus, the novel genetic influences for reading achievement at each age in the study by Harlaar and colleagues might correspond to the novel genetic variance in the current study, but be larger in magnitude because the emphasis of teacher-rated reading scores likely varies across ages and represent a wider range of reading skills (i.e., beyond just reading comprehension) at each year of evaluation. Across studies, there is evidence for common genetic influences on reading and reading-related skills. Given the current findings that show stable genetic influences on reading comprehension, it seems as though there is a core of genetic effects for reading comprehension that persist across reading development. This core of genetic effects is likely comprised of reading-related skills identified in extant research, and a better understanding of these core influences with regard to the relative importance and timing of the underlying mechanisms, if known, could be informative to better target intervention efforts.

Turning to the environment, shared environmental effects were more modest and only significant at grades 1 and 2. These results were in line with the effect sizes expected given extant findings for reading, see [25]. Though it is well-established in the phenotypic literature that environmental factors influence mean levels of reading, the shared environment was not contributing significantly to variability in reading comprehension after the early school years. Instead, stability in reading development was largely mediated by continuous genetic influences. These results should not be interpreted to mean that the shared environment has minimal impact on mean reading comprehension outcomes, but rather that the shared environment overall was not affecting variation in individual differences. That is, differences in the shared environment were not driving differences in reading comprehension after second grade. Finally, the nonshared environmental influences were mostly novel at each grade. These grade-specific effects may include differential effects of particular classrooms and teachers as children change classes from year to year, and measurement error.

In addition to noting that we are addressing individual differences rather than mean levels of performance, the current findings should be considered in light of some limitations. Potential limitations of most behavioral genetic studies with identical and fraternal twins are their assumptions that all genetic influence is additive (i.e., differences are a result of additive allelic substitutions in contrast to dominance or interactive allelic effects), and that there is no assortative (non-random) mating in which the similarity of mates is greater than expected by chance. Assortative mating can impact the relative modeled estimates of genetic and environmental influences for the studied phenotype. There is evidence against both assumptions for other phenotypes such as personality [40]. Violation of the additivity assumption can lead to an underestimate of shared environment influences and an overestimate of genetic influences. While non-additivity cannot be completely ruled out, no direct evidence for violation of the additivity assumption for reading comprehension (i.e., significant negative shared environmental effects, or DZ twin correlations less than half those of MZ twins—necessary but not sufficient to rule out non-additivity) was observed. Another limitation of the current study is that reading was assessed using a single measure. Reading comprehension is a complex skill and Keenan and colleagues have demonstrated that different reading comprehension measures differ in their relative dependence on decoding versus oral language comprehension [41] and can influence the outcomes of genetic analyses [42]. Though the current reading comprehension measure is highly reliable and widely used, it relies somewhat more heavily on decoding skills relative to oral language comprehension, thus, results should be considered in light of this context. Betjemann and colleagues [42] showed test differences on genetic influences such that reading comprehension tests that used more extended discourse showed distinct genetic influences on decoding and comprehension. Future research using such tests would allow us to determine the extent to which stability of genetic influences on reading comprehension stem from decoding, or whether there are additional, separate genetic influences from comprehension.

The aforementioned limitations notwithstanding, these results are important because they suggest that genetic influences are not only significant, accounting for nearly 75% of the total variance in reading comprehension, but they are also largely stable. There are several important implications for educational theory and practice. First, mean changes in reading comprehension are clearly due primarily to instruction, but changes in individual differences in reading comprehension within and across ages are largely genetic. Thus, there may be a core of genetic influences that pervade reading development and are resistant to differences in the environment. Another possibility is that the increasing reliance on standardized instruction across schools has homogenized and reduced the amount of environmental variance, leaving genetic differences as a relatively more important factor in explaining individual differences. It is important to emphasize that sample differences in environmental range will likely influence estimates of genetic and environmental influences. For example, there is direct evidence that large environmental variation in reading instruction will lead to higher estimates of shared environment and lower estimates of genetic influences [25].

Interpretation of the large and stable genetic effects is tempered by the fact that some observed genetic and environmental stability may result from gene-environment correlations. This occurs to the extent that the same genetic factors influencing reading comprehension also actively shape environmental conditions that create stability in future reading. Children’s genetically-influenced reading skill levels might, for example, lead them to seek out more or less independent reading (active gene-environment correlation) or increase the probability that adults direct more or fewer resources towards their instruction (evocative gene-environment correlation). Only one known study of reading has attempted to examine this question. Using the same sample of Ohio twins described in this report, an investigation of the relation between reading comprehension and independent reading from age 10 to 11 found that while reading comprehension skill predicted subsequent independent reading, independent reading did not predict subsequent reading skill [43]. Although for this limited time range independent reading does not seem to be contributing to stability in reading comprehension, it is quite possible that a reciprocal relation exists at other developmental stages. Such reciprocal relations are not well understood, and more longitudinal studies with independent reading as well as a wider range of environmental variables which might be affecting stability in reading comprehension are needed to identify potential opportunities for prevention and intervention of reading difficulties.

In closing, reading comprehension is a complex skill involving the coordination of many cognitive and language abilities. Current findings have implications for cognitive scientists interested in understanding the etiological underpinnings of cognitive processes as well as educators concerned with reading development. Although it is clear that reading skill is influenced by environmental factors, the current findings demonstrate that there are substantial heritable factors underlying reading comprehension. Further, these genetic influences appear to be stable across a broad range of development. It is therefore important to recognize these heritable influences and consider their potential to impact delivery of instruction and intervention.
